# High prevalence of and factors associated with human papillomavirus infection among women attending a tertiary hospital in Gauteng Province, South Africa

**DOI:** 10.1186/s12885-022-09964-9

**Published:** 2022-08-05

**Authors:** Teboho Amelia Tiiti, Selokela Gloria Selabe, Johannes Bogers, Ramokone Lisbeth Lebelo

**Affiliations:** 1 Department of Virological Pathology, Sefako Makgatho Health Sciences University, Pretoria, South Africa; 2 Laboratory of Cell Biology and Histology, Faculty of Medicine and Health Sciences, University of Antwerp, Antwerp, Belgium; 3 Department of Virological Pathology, National Health Laboratory Service, Sefako Makgatho Health Sciences University, Pretoria, South Africa; 4 Applied Molecular Biology Research Group (AMBIOR), University of Antwerp, Antwerp, Belgium; 5 South African Vaccination and Immunization Centre, Sefako Makgatho Health Sciences University, Pretoria, South Africa

**Keywords:** Human papillomavirus, E6/E7 mRNA, Prevalence, Risk factors, HPV 16, HPV 18

## Abstract

**Background:**

Persistent high-risk (hr) human papillomavirus (HPV) infection is a necessary cause of cervical cancer. Cervical cancer is a major public health problem in Sub-Saharan Africa including South Africa. This study investigated the prevalence of and factors associated with hr-HPV infection among women attending a tertiary hospital in Gauteng Province, South Africa.

**Methods:**

Cervical samples were collected from 526 participants aged ≥ 18 years using a Cervex Brush® Combi and tested for hr-HPV types on the Abbott m2000 analyzer using the Abbott RealTime HR HPV assay. Samples that tested hr-HPV deoxyribonucleic acid (DNA)-positive were further tested for hr-HPV E6/E7 messenger ribonucleic acid (mRNA) using the APTIMA® HPV assay on the Panther system (Hologic, Inc.). Sociodemographic data were collected using a self-administered questionnaire. Binomial regression analysis was used to assess factors associated with hr-HPV infection.

**Results:**

Overall hr-HPV DNA prevalence was 48.1% (95%CI: 43.8–52.4%). Of the hr-HPV DNA-positives, 24.5% (95%CI: 19.3–30.1) had HPV-16; 12.3% (95%CI: 8.5–16.9) had HPV-18 and 87.4% (95%CI: 82.6–91.2) had other 12 h-HPVs. Of the samples positive for hr-HPV DNA, 84.2% (95%CI: 79.1–88.5) (213/253) were positive for hr-HPV E6/E7 mRNA. Advanced age was an important factor linked to hr-HPV E6/E7 mRNA positivity. Based on multivariate binomial regression analysis, unemployment (PR: 1.50; 95%CI: 1.23–1.83) and being married (PR: 0.61; 95%CI: 0.47–0.81) were identified as statistically significant (*p* < 0.0001) predictive and protective factors, respectively, for hr-HPV infection.

**Conclusions:**

The prevalence of hr-HPV infection was high. Furthermore, hr-HPV DNA-positive samples had a high hr-HPV E6/E7 mRNA prevalence. The presence of hr-HPV E6/E7mRNA indicates active infection and thus a greater risk of developing the cervical disease. Therefore, HPV mRNA testing could be a better test to monitor women who are positive with Pap smear before colposcopy is performed to reduce the burden of referrals.

**Supplementary Information:**

The online version contains supplementary material available at 10.1186/s12885-022-09964-9.

## Background

Worldwide, cervical cancer is one of the most common cancers in women; with 604,127 new cases and 341,831 deaths reported in 2020 [1]. Sub-Saharan African regions including South Africa have experienced increasing trends ranging from 2 to 10% over 20 years [1]. Furthermore, cervical cancer cases are most common among women between the ages of 15 to 44 years) [2]. High-risk human papillomavirus (hr-HPV) is the most common sexually transmitted infection (STI) [3]; and an etiologic agent for the development of cervical cancer [4]. Although 70% of sexually active individuals are infected with HPV, the infection usually clears up within a few months, with 90% of cases clearing within two years after HPV acquisition [5]. However, 10 to 20% of HPV infections persist latently, leading to disease progression and eventually invasive cancer [6]. HPV messenger ribonucleic acid (mRNA) tests determine whether the virus is actively expressing the proteins responsible for oncogenesis in HPV infection [7] and thus allow the identification of transient and potentially transforming HPV infections [8]. E6 and E7 oncogene expression cause cellular changes [9]. Cellular changes are achieved when genes hijack the cellular ubiquitin–proteasome system (UPS) to degrade or inactivate the cellular tumor suppressor protein p53 and the retinoblastoma protein (pRb), forcing the host cell into the S phase [10].

To curb cervical cancer incidence and mortality rates in South Africa, two HPV vaccines; Cervarix® (GlaxoSmithKline Biologicals, Rixensart, Belgium) and Gardisil® (Merck & Co., Whitehouse Station, NJ USA) were approved in 2008 [11]. In 2014, the first-dose phase of the South African National HPV campaign was launched through the Integrated School Health Programme implemented by the National Department of Health (NDoH), in collaboration with the Department of Basic Education targeting female learners aged 9 years and older in Grade 4 [12]. Cervarix® protects against two hr-HPV types, HPV-16, and HPV-18, which together account for 70% or more of all cervical cancers globally [13]. Gardisil® confers protection against two low-risk (lr) HPV types causing about 90% of warts (HPV-6 and HPV-11) and two hr-HPV types (HPV-16 and HPV-18). Although the approved vaccines do not prevent diseases caused by other hr-HPV types, it is anticipated that they will contribute to significant reductions in cervical cancer incidence in the country. Therefore, effective cervical cancer screening will remain the most important secondary-level prevention intervention.

Advances in cervical cancer screening technologies have introduced HPV testing due to the higher sensitivity for precancer and cancer detection than cytology [14]. In primary HPV screening programs, two types of hr-HPV assays are used; HPV tests that are very sensitive for HPV deoxyribonucleic acid (DNA) detection and HPV-specific tests that target HPV E6/E7 oncogenes with high specificity but lower sensitivity [15]. Although HPV testing is replacing cytology, particularly in HICs, the test is associated with lower specificity, which hence requires a second test to identify women who are at risk of developing cervical cancer [16]. The low specificity associated with HPV DNA testing may lead to unnecessary colposcopy referral and overtreatment, hence some countries tailor the triage strategy and/or follow-up [17].

HICs routinely use cervical cytology and HPV testing to detect high-grade intraepithelial neoplasia to prevent invasive cervical cancer. However, in low and middle-income countries (LMICs) there is a limitation of resources such as health infrastructure, and trained personnel, which therefore hinder the feasibility and effectiveness of Papanicolaou (Pap) smear tests [18]. The lack of adequate infrastructure may also pose a challenge to the introduction of liquid-based cytology (LBC). Nevertheless, some LMICs have introduced HPV testing in their national cervical cancer screening programs [17]. In South Africa, in addition to LBC-based cervical cancer screening, the NDoH Cervical Cancer Prevention and Control Policy has recommended HPV DNA-based testing in the public sector [19].

The acquisition and persistence of HPV infection and development of HPV-associated cancers are associated with high-risk sexual behavior, such as the age of first vaginal sex, age of first oral sex, and the number of oral and vaginal sexual partners [20]. Other risk factors of HPV infection include STIs such as HIV (Swaziland) [21]; being single and cohabiting with a sexual partner (Ghana) [22]; practicing intravaginal substance use (South Africa) [23]; and an increased number of lifetime sexual partners (South Africa) [24].

Hr-HPV testing is relatively new to sub-Saharan Africa (SSA), and most studies have utilized hr-HPV DNA assays compared with hr-HPV E6/E7 mRNA assays in the region. Although data on HPV DNA prevalence and associated factors are available in SSA regions including South Africa, there is a lack of data on the prevalence and associated factors of hr-HPV E6/E7 mRNA in the country. Furthermore, despite the high burden of cervical cancer in the country, there is limited research on factors associated with hr-HPV E6/E7 mRNA expression. Therefore, this study aimed to investigate the prevalence of and factors associated with hr-HPV infection among women attending tertiary hospital gynecology clinics in Gauteng Province, South Africa.

## Methods

### Study design and population

This was a cross-sectional study conducted from March 2016 to September 2018 at a tertiary hospital, situated in the north of Pretoria in the Gauteng Province, South Africa. The study participants were women aged 18 years and older, attending gyne-oncology and termination of pregnancy (TOP) clinics within the tertiary hospital. All women who had provided written informed consent were included. Those who had undergone a complete hysterectomy or were menstruating at the time of enrolment were excluded. Services offered at the gyne-oncology clinic included large loop excision of the transformation zone (LLETZ) procedure, a review with Pap test after treatment with LLETZ, and colposcopy examination after a positive Pap test. The termination of pregnancy (TOP) clinic under the gynecology department offered family planning, cervical cancer screening using the Pap test, and other gynecology-related services.

### Ethical approval and consent to participate

The study was conducted with the approval of the Research Ethics Committee of Sefako Makgatho Health Sciences University, (SMUREC/P/75/2016: IR) and (SMUREC/M/279/2019: PG). Written informed consent was signed by each participant.

### Data and specimen collection

Women were provided with written information sheets that had a brief description and purpose of the study. Consenting participants completed a self-administered questionnaire covering demographic characteristics, as well as information related to sexual practices and reproductive history, knowledge of HPV, cervical cancer, and participants’ experiences and preferences using the self-sampling device before clinical examination for cervical sample collection. The researchers distributed the questionnaire after the study had been explained to the participants.

An endo/ectocervical sample was collected using a Cervex Brush® Combi (Rovers® Medical Devices B.V., the Netherlands). The speculum was inserted and opened to widen the vagina to assist the pelvic examination, and thereafter, the brush was delicately inserted into the endocervical canal until the lateral bristles were touching the ectocervix and rotated two full turns clockwise. The collection brushes were immediately rinsed into ThinPrep PreservCyt® Solution vials (Hologic Incorporated, Bedford, Massachusetts) for the preservation of collected cells, before being discarded. For laboratory testing, all samples were transported at room temperature to the HPV and STIs Training Centre for Africa, Department of Virology at the Sefako Makgatho Health Sciences University.

### Detection of high-risk-HPV DNA

The Abbott RealTime HR HPV assay (Abbott Molecular GmbH & Co. KG, Wiesbaden, Germany) is a qualitative fully automated assay detecting 14 h-HPV types (16, 18, 31, 33, 35, 39, 45, 51, 52, 56, 58, 59, 66 and 68) from ThinPrep PreservCyt collected samples. The assay genotypes only HPV-16 and HPV-18, while the other 12 h-HPV types were reported as a pooled result “Other hr-HPV”. Hr-HPV DNA extraction, amplification, and genotyping were conducted according to the manufacturer's instructions.

### Detection of hr-HPV E6/E7 mRNA

Samples that tested positive for hr-HPV DNA with the Abbott m2000 system were tested for hr-HPV E6/E7 messenger ribonucleic acid (mRNA) using the APTIMA® HPV assay (Hologic Gen-Probe, Inc., San Diego, Canada). This assay is an in vitro nucleic acid amplification test for the qualitative detection of hr-HPV E6/E7 viral mRNA of the same 14 h-HPV types detected by the Abbott RealTime HR HPV assay. An aliquot of 1 mL of each Thin Prep PreservCyt vial was diluted into 2.9 mL of Aptima specimen transport media and tested according to the manufacturer's instructions. The assay incorporated an internal control (IC) to monitor nucleic acid capture, amplification, and detection, as well as operator or instrument errors. The results were interpreted as negative, positive, or invalid if the run or IC failed. The results did not discriminate between the 14 h-HPV types, with the detection of any of the 14 h-HPV types showing a positive result.

### Statistical analysis

Statistical analysis was performed using SAS version 9.4 for Windows. Descriptive data analyses included the mean, standard deviation (SD), the range for age, and frequency distributions for categorical data. Prevalence estimates were calculated by dividing the number of infected individuals by the total number of the sample. Exact binomial 95% Confidence intervals (CIs) were calculated for proportions. The Prevalence Ratios (PRs) of each risk factor for each outcome variable (hr-HPV infection and hr-HPV E6/E7 mRNA expression) were determined, together with its 95% confidence interval, using binomial regression. Categories with *n* < 15 overall were not included in analyses (no reliable inference can be made based on such small groups). Study variables significant at *p* < 0.20 were combined into a multivariable model, after examining each pair of variables for possible confounding using the chi-squared test (or Fisher’s exact test for 2 × 2 tables). All variables (with *p* < 0.20) were at first included in the multivariate model, but after sequentially removing those which were not significant. A value of Cramer’s V (or the phi coefficient for Fisher’s exact test) > 0.60 was regarded as too strong an association to include both variables in a multivariable model. In the multivariable model, non-significant variables were removed one at a time until only significant variables remained. A p-value of ≤ 0.05 was considered statistically significant.

## Results

### Demographic characteristics

The mean age of participants was 36.8 (SD: ± 11.04; range: 18 to 68 years. The majority (61.6% [322/523]) of the participants were between the ages of 18 to 39 years. All 526 enrolled women were black Africans (99.6%), except for two women (0.4%). The majority (67.3%) of the participants were single. The majority (85.6%) of participants were from semi-urban (township) areas compared to only 2.3% from rural areas. More than fifty percent (57.4%) of the participants had 1 or 2 children and most (79.8%) of the participants were sexually active with 74.5% having at least one sexual partner at the time of the study. Of the 526 participants, few (28.9%) reported the use of contraceptives, only 17.1% were pregnant, and 16.0% of pregnancies were in their first trimester Table**1**.

**Table 1:** Demographic characteristics and overall hr-HPV prevalence of the study population

	***n***	**%**
Age (years) (*n* = 523)		
18–29	151	28.9
30–39	171	32.7
40–49	121	23.1
50–59	61	11.7
60 +	19	3.6
Marital status (*n* = 526)		
Single	354	67.3
Married	126	24.0
Divorced/Widowed/Separated	46	8.7
Employment status (*n* = 526)		
Employed	237	45.1
Unemployed	289	54.9
Place of residence (*n* = 522)		
Rural	12	2.3
Semi-urban	448	85.6
Urban	63	12.1
Number of children (*n* = 526)		
None	92	17.5
1–2	302	57.4
3–4	118	22.4
5 and more	14	2.7
Present sexual activity status (*n* = 526)		
Not active	106	20.2
Active	420	79.8
Number of current sexual partners (*n* = 526)		
None	100	19.0
1	392	74.5
2 or more	34	6.5
Number of past sexual partners over 12 months (*n* = 526)		
None	99	18.8
1	214	40.7
2	76	14.4
3	67	12.7
4 or more	70	13.3
Use of contraceptives (*n* = 526)		
Not using	374	71.1
Using	152	28.9
Period of contraceptive use (*n* = 526)		
6 months	33	6.3
12 months	25	4.8
24 months	34	6.5
More than 24 months	60	11.4
N/A	374	71.1
Pregnant (*n* = 526)		
Not pregnant	427	81.2
Pregnant	90	17.1
Don’t know	9	1.7
Period of pregnancy (*n* = 526)		
1–3 months	84	16.0
4–6 months	5	1.0
7–9 months	1	0.2
N/A	436	82.9
Hr-HPV		
Hr-HPV DNA	253	48.1
HPV type 16	62	24.5
HPV type 18	31	12.3
Other 12 h-HPVs	221	87.4
Hr-HPV E6/E7 mRNA (*n* = 253)	213	84.2

Note that n adds up to more than 253 because of multiple hr-type infections in some samples

### Prevalence of hr-HPV infection

Of all participants, 48.1% (253/526) were hr-HPV DNA-positive, with HPV-16, HPV-18, and the other 12 h-HPV types being positive in 11.8% (62/526), 5.9% (31/526) and 42.0% (221/526), respectively. Hr-HPV types targeted by the bivalent HPV vaccine (HPV-16/-18) were detected in 15.4% (81/526) of participants. HPV-16 and HPV-18 were detected in 2.3% (12/526) of participants. Table 1 summarizes the results for all studied samples.

### Distribution of hr-HPV types and hr-HPV E6/E7 mRNA

Among those with hr-HPV infection, HPV-16 (8.7%) was prevalent compared to HPV-18 (3.2%). Additionally, of all positive samples, 32.0% (81/253) were positive for either HPV-16 or HPV-18 or both HPV-16 and HPV-18. Among those with multiple hr-HPV infections, HPV-16 + other 12 h-HPVs combination (11.1%) was the most common Table 2. Hr-HPV E6/E7 mRNA prevalence in samples positive for HPV-16 and HPV-18 only was 95.4% (21/22) and 75.0% (6/8), respectively. In samples positive for the other 12 h-HPVs (non-HPV-16/-18), hr-HPV E6/E7 mRNA prevalence was 79.6% (137/172).

**Table 2:** Frequency distribution of hr-HPV types and hr-HPV E6/E7 mRNA

Hr-HPV types	Hr-HPV -positive n (%) *n* = 253	95%CI	Hr-HPV E6/E7 mRNA positive *n* (%)	95%CI
**Single Test Positive only**				
HPV-16 only	22 (8.7)	5.2–12.2	21 (95.4)	77.1–99.9
HPV-18 only	8 (3.2)	1.0–5.3	6 (75.0)	34.9–96.8
Other 12 h-HPVs only	172 (68.0)	62.2–73.7	137 (79.6)	72.8–85.4
**Multiple Tests Positive only**				
HPV-16 + 18	2 (0.8)	0.1–2.8	2 (100)	15.8–100
HPV-16 + Other 12 h-HPVs	28 (11.1)	7.2–14.9	27 (96.4)	81.6–99.9
HPV-18 + Other 12 h-HPVs	11 (4.3)	1.8–6.9	11 (100)	71.5–100
HPV-16 + 18 + Other 12 h-HPVs	10 (4.0)	1.6–6.4	9 (90.0)	55.5–99.7

### Prevalence of hr-HPV types and E6/E7 mRNA in different groups

Hr-HPV DNA and hr-HPV E6/E7 mRNA among participants attending gynecology clinics were stratified by reasons for clinic visits. There was a significant moderate association between hr-HPV infection and reason for visit (*p* < 0.0001; Cramer's V = 0.33). The prevalence of hr-HPV DNA and hr-HPV E6/E7 mRNA was significantly higher for those who visited the clinic for the LLETZ procedure and significantly lower for those who came for a routine Pap smear and termination of pregnancy (TOP) Table 3. There was a significant but weak association between hr-HPV E6/E7 mRNA expression and reasons for a visit (*p* = 0.0001; Cramer's V = 0.29).

**Table 3:** Prevalence of hr-HPV types and hr-HPV E6/E7 mRNA in women attending gynecology clinics for different reasons

		Overall		Overall hr-HPV DNA prevalence		***p***-value for between the -group test	Overall hr-HPV E6/E7 mRNA prevalence		***p***-value for between the -group test
Characteristic	Category	n	%	n	row %		n	row %	
**n**		**526**		**253**	48.1		**213/253**	84.2	
Reason for visit	Routine Pap smear	232	44.1	78	33.6	<0.0001 (V=0.33)	58/78	74.4	0.0001 (V=0.29)
	LLETZ	87	16.5	69	79.3		67/69	97.1	
	Termination of pregnancy	87	16.5	46	52.9		34/46	73.9	
	Review with Pap smear after LLETZ	57	10.8	30	52.6		26/30	86.7	
	Colposcopy	33	6.3	18	54.5		18/18	100.0	
	Family planning	30	5.7	12	40.0		10/12	83.3	

### Age-specific prevalence of hr-HPV DNA and hr-HPV E6/E7 mRNA

There was a high hr-HPV DNA prevalence (57.0%) in women younger than 30 years of age. Hr-HPV DNA prevalence decreased (45.6%) in women aged 30 to 39 years and increased (48.8%) aged 40 to 49 years. Hr-HPV prevalence then decreased with increasing age Table 4. For hr-HPV E6/E7 mRNA, the prevalence was high in women over 40 years. In contrast to hr-HPV DNA, hr-HPV E6/E7 mRNA was lower in women younger than 30 years Table 5.

**Table 4:** Prevalence of and factors associated with hr-HPV infection

Characteristics	Total	HR-HPV prevalence	Bivariate analysis		Multivariate analysis	
	***n***	***n* (%)**	**PR (95% CI)**	***p*-value**	**PR (95% CI)**	***p*-value**
Age (years)						
18–29	151	86 (57.0)	1.00			
30–39	172	78 (45.6)	0.80 (0.65–0.99)	**0.043**		
40–49	121	59 (48.8)	0.86 (0.68–1.08)	0.18		
50–59	61	23 (37.7)	0.66 (0.47–0.94)	**0.021**		
60 +	19	7 (36.8)	0.65 (0.35–1.18)	0.16		
Marital status						
Single	354	196 (55.4)	1.00		1.00	
Married	126	39 (31.0)	0.56 (0.42–0.74)	** < 0.0001**	0.61 (0.47–0.81)	**0.0006**
Divorced/Widowed/Separated	46	18 (39.1)	0.71 (0.49–1.03)	**0.068**	0.79 (0.54–1.14)	0.20
Employment status						
Employed	237	85 (35.9)	1.00		1.00	
Unemployed	289	168 (58.1)	1.68 (1.33–1.97)	** < 0.0001**	1.50 (1.23–1.83)	** < 0.0001**
Place of residence						
Rural	12	5 (41.7)	0.85 (0.43–1.66)	0.63		
Semi-urban	447	220 (49.2)	1.00			
Semi-rural	63	28 (44.4)	0.90 (0.67–1.21)	0.49		
Number of children						
None	92	44 (47.8)	1.00			
1 or 2	302	148 (49.0)	1.02 (0.80–1.31)	0.84		
3 or 4	118	56 (47.5)	0.99 (0.75–1.32)	0.96		
5 and more	14	5 (35.7)	0.75 (0.36–1.56)	0.44		
Present sexual activity status						
Not active	106	57 (53.8)	1.00			
Active	420	196 (46.7)	0.87 (0.71–1.06)	0.17		
Number of current sexual partners						
None	100	55 (55.0)	1.00			
1	392	178 (45.4)	0.83 (0.67–1.02)	0.071		
2 or more	34	20 (58.8)	1.07 (0.77–1.49)	0.69		
Number of past sexual partners over 12 months						
None	99	52 (52.5)	1.00			
1	214	91 (42.5)	0.81 (0.63–1.03)	0.089		
2	76	43 (56.6)	1.08 (0.82–1.41)	0.59		
3	67	32 (47.8)	0.91 (0.67–1.24)	0.55		
4 or more	70	35 (50.0)	0.95 (0.71–1.28)	0.75		
Use of contraceptives						
Not using	374	180 (48.1)	1.00			
Using	152	73 (48.0)	1.00 (0.82–1.21)	0.98		
Period of contraceptive use						
6 months	33	19 (57.6)	1.20 (0.88–1.63)	0.26		
12 months	25	10 (40.0)	0.83 (0.51–1.36)	0.46		
24 months	34	16 (47.1)	0.98 (0.67–1.42)	0.91		
more than 24 months	60	28 (46.7)	0.97 (0.73–1.30)	0.84		
N/A	374	180 (48.1)	1.00			
Pregnant						
Not pregnant	427	202 (47.3)	1.00			
Pregnant	90	46 (51.1)	1.08 (0.86–1.35)	0.50		
Don’t know	9

**Table 5:** Prevalence of and factors associated with hr-HPV E6/E7 mRNA expression

Characteristics	Total	Hr-HPV E6/E7 mRNA prevalence	Bivariate analysis		Multivariate analysis	
	***n***	***n* (%)**	**PR (95% CI)**	***p*-value**	**PR (95% CI)**	***p*-value**
Age (years)						
18–29	86	60 (69.8)	1.00		1.00	
30–39	78	69 (88.5)	1.27 (1.08–1.49)	**0.0038**	1.27 (1.08–1.49)	**0.0038**
40–49	59	57 (96.6)	1.38 (1.20–1.60)	** < 0.0001**	1.38 (1.20–1.60)	** < 0.0001**
50–68	30	27 (90.0)	1.29 (1.07–1.55)	**0.0065**	1.29 (1.07–1.55)	**0.0065**
Marital status						
Single	196	162 (82.7)	1.00			
Married	39	33 (84.6)	1.02 (0.89–1.16)	0.80		
Divorced/widowed/separated	18	18 (100)	Not estimable			
Employment status						
Employed	85	71 (83.5)	1.00			
Unemployed	168	142 (84.5)	1.01 (0.90–1.13)	0.84		
Place of residence						
Semi-urban	220	183 (83.2)	1.00			
Semi-rural/Rural	33	30 (90.9)	1.09 (0.97–1.24)	0.16		
Number of children						
None	44	33 (75.0)	1.00			
1 or 2	148	126 (85.1)	1.14 (0.94–1.3)	0.18		
3 or more	61	54 (88.5)	1.18 (0.97–1.43)	0.092		
Present sexual activity status						
Not active	57	47 (82.5)	1.00			
Active	196	166 (84.7)	1.03 (0.90–1.17)	0.69		
Number of current sex partners						
None	55	47 (85.5)	Ref			
1	178	149 (83.7)	0.98 (0.86–1.11)	0.75		
2 or more	20	17 (85.0)	0.99 (0.80–1.23)	0.96		
Number of past sexual partners over 12 months						
None	52	45 (86.5)	1.00			
1	91	70 (76.9)	0.89 (0.76–1.04)	0.14		
2	43	38 (88.4)	1.02 (0.88–1.19)	0.79		
3	32	27 (84.4)	0.98 (0.81–1.17)	0.79		
4 or more	35	33 (94.3)	1.09 (0.95–1.25)	0.21		
Use of contraceptives						
Not using	180	149 (82.8)	1.00			
Using	73	64 (87.7)	1.06 (0.95–1.18)	0.30		
Period of contraceptive use						
6 – 24 months	45	37 (82.2)	0.99 (0.85–1.16)	0.93		
More than 24 months	28	27 (96.4)	1.16 (1.06–1.28)	0.0022		
N/A	180	149 (82.8)	1.00			
Pregnant						
Not pregnant	202	174 (86.1)	1.00			
Pregnant	46	34 (73.9)	0.89 (0.77–1.04)	**0.147**		
Don’t know	5					

### Factors associated with hr-HPV infection

Table 4 shows factors associated with hr-HPV infection. Bivariate analysis revealed a significant association between hr-HPV infection with age, marital, and employment status. Being 50 to 59 years [Prevalence Ratio (PR): 0.66; 95% confidence interval (CI): 0.47–0.94; *p* = 0.021)] and being married (PR: 0.56; 95% CI: 0.42–0.74; *p* < 0.0001) were statistically significant protective factors, whereas being unemployed (PR: 1.62; 95% CI: 1.33–1.97; *p* < 0.0001) was a predictive factor for hr-HPV infection. Even after multivariable analysis, unemployment and being married were statistically significant predictive and protective factors, respectively, for hr-HPV infection.

### Factors associated with hr-HPV E6/E7 HPV expression

Based on the bivariate analysis, age was significantly associated with hr-HPV E6/E7 mRNA expression. Being between the ages of 40 to 49 years was significantly associated with an increased risk (PR: 1.38; 95% CI: 1.20–1.60; *p* < 0.001) of hr-HPV E6/E7 mRNA expression. Furthermore, contraceptive use for more than 24 months was a (PR: 1.16; 95% CI: 1.06–1.28; *p* = 0.0022) was a predictive factor for hr-HPV E6/E7 mRNA. Multivariate binomial regression identified age as a statistically significant predictive factor for hr-HPV E6/E7 mRNA Table 5.

### Factors associated with hr-HPV infection in women attending gynecology clinics for different reasons

Bivariate analysis revealed a significant association between hr-HPV infection with age, marital, and employment status in women attending routine Pap smear, Advanced age was statistically not associated with hr-HPV infection; 30–39 (PR: 0.58; 95CI: 0.38–0.88; *p* = 0.010); 40–49 (PR: 0.53; 95%CI: 0.33–0.86; *p* = 0.010) and 50–59 (PR: 0.37; 95%CI: 0.18–0.75; *p* = 0.006). Being married (PR: 0.39; 95% CI: 0.23–0.66; *p* = 0.0004) and divorced/widowed/separated (PR: 0.47; 95%CI: 0.23–0.98) were statistically significant protective factors, whereas being unemployed (PR: 2.26; 95% CI: 1.59–3.22; *p* < 0.0001) was a predictive factor for hr-HPV infection. In a multivariate binomial regression, only marital status and employment status remained significantly associated with hr-HPV infection Table S1.

### Factors associated with hr-HPV E6/E7 mRNA expression in women attending gynecology clinics for different reasons

Bivariate analysis revealed a significant association between hr-HPV E6/E7 mRNA expression and age among women who attended routine Pap smear. Advanced age, 40–49 (PR: 1.49; 95%CI: 1.09–2.04; *p* = 0.014) and 50–68 (PR: 1.44; 95%CI: 1.02–20.04; *p* = 0.037) were statistically associated with hr-HPV E6/E7 mRNA expression. In a multivariate model, only age remained significantly associated with hr-HPV E6/E7 mRNA expression Table S2.

## Discussion

The current study used the Abbott RealTime HR HPV assay and the APTIMA® HPV assay to detect 14 h-HPV types and the expression of hr-HPV E6/E7 mRNA of these HPV types among women attending gynecology clinics at a tertiary hospital in Gauteng Province, South Africa from 2016 and 2018. The overall hr-HPV DNA prevalence was 48.1%. Comparable hr-HPV DNA prevalence was reported in previous South African studies; 54.5% among HIV-negative women aged 14 to 30 years [23] and 54.3% among women attending public sector primary healthcare clinics for routine gynecological and non-gynecological primary healthcare-related reasons [25], respectively. Similar HPV prevalence was reported by Ginindza et al. [21] in Swaziland (46.2%) and Mbulawa et al. [24] in South Africa (45.7%) using the GeneXpert HPV assay among women aged 15 to 49 years, and the Roche Linear Array HPV genotyping test among HIV-negative women aged 16 to 22 years, respectively. Low HPV DNA prevalence (28.5%) compared to our study was found among 30 to 98 years HIV-positive and -negative women recruited from a community health clinic in South Africa [26]. The relatively low HPV DNA prevalence reported by Taku et al. [26] could be influenced by the exclusion of the younger population that has been shown to harbor higher HPV infection. Although HPV DNA prevalence is higher among young women, most infections are transient and asymptomatic [27]; therefore, clearing in the majority and only persisting in the minority.

High hr-HPV DNA prevalence (57.0%) among the younger population aged 18–29 years in the current study supports the continuation of HPV vaccination for South African girls. Consistent with previous studies, a high prevalence of HPV DNA among young women has previously been reported in Swaziland, South Africa, and the Democratic Republic of Congo [21, 23, 28–30]. The second peak of HPV DNA prevalence in the group of women aged 40 to 49 years old observed in the current study was previously reported in the same age group in Korea [31]. Previously, other studies have observed a second peak of prevalence in the group of women aged 46 to 66 and 60 years and older, respectively [28, 29]. The second peak of prevalence in the current study might be explained by the low cervical cancer screening coverage observed among the study population.

The current study also demonstrates a substantially higher hr-HPV E6/E7 mRNA prevalence than what was found by other studies within the African continent. Hr-HPV E6/E7 mRNA prevalence in our study was higher compared to the overall hr-HPV E6/E7 mRNA of 36.7% reported among HIV-infected women in South Africa and 30.0% among sex workers in Kenya [32, 33]. The abovementioned studies tested all samples as opposed to the current study that only tested hr-HPV DNA-positive samples; this could explain the high prevalence found in our study. Comparable to our study, a previous study reported a slightly lower hr-HPV E6/E7 mRNA prevalence (67%) among HPV DNA-positive women [9]. The high hr-HPV DNA and hr-HPV E6/E7 mRNA prevalence in the current study is likely due to the high-risk nature of the study population, particularly women who were scheduled for the LLETZ procedure, colposcopy, and those who had already undergone the LLETZ procedure. In the current study, not all women who tested positive for hr-HPV DNA expressed hr-HPV E6/E7 mRNA. The detection of HPV E6/E7 mRNA is suggestive of an increased risk of cervical cancer development as it shows active or progressing HPV infection [34]. Additionally, HPV E6/E7 mRNA detection is more specific than HPV DNA in identifying women at risk of cervical disease [27]. In the current study, hr-HPV E6/E7 mRNA prevalence increased with age, where women aged ≥ 30 years had the highest prevalence and those < 30 years had the lowest prevalence, contrary to hr-HPV DNA. In the current study, hr-HPV E6/E7 mRNA prevalence increased with age, where women aged ≥ 30 years had the highest prevalence and those < 30 years had the lowest prevalence, contrary to hr-HPV DNA. Although HPV infection is very high in younger women; the infections are transient and asymptomatic [27]. However, advanced age is one of the factors for the persistence of HPV infection [35]; which results from increased E6/E7 mRNA expression [36]. In advanced age groups, a high prevalence of hr-HPV mRNA has been previously reported [37].

The overall HPV-16 prevalence accounted for 11.8% (62/526) of samples. This finding is comparable to 12.4% previously reported among women 15 to 49 years attending healthcare facilities in Swaziland [21], and 16.0% among women 18 to 68 years attending obstetrics and gynecology clinics in Ethiopia [38]. The prevalence of HPV-16/-18 (15.4%; 81/526) is comparable to those previously reported in South African studies, 18.6% and 19.5% [24, 25]. In this study, hr-HPV DNA type distribution showed that an aggregate of the other 12 h-HPV types dominated over HPV types 16 and 18, a finding that is consistent with previously reported studies in Ethiopia and Botswana using the Abbott HR HPV assay, respectively [38, 39]. The other 12 h-HPV types (31, 33, 35, 39, 45, 51, 52, 56, 58, 59, 66, and 68), accounted for 87.4% of cases, a finding (76.0%) that is consistent with a previous study in Ethiopia [38].

As expected, a high prevalence of hr-HPV infection and expression of hr-HPV E6/E7 mRNA was found among women undergoing the LLETZ procedure and colposcopy. Factors including smoking, hr-HPV type, and persistence of hr-HPV posttreatment amongst others may contribute to treatment failure [40]; thus, patients are followed up following the LLETZ procedure. Hr-HPV DNA prevalence was high in women who had the LLETZ procedure. This is expected as HPV infection is still present in up to one-third of women who had undergone the procedure [41]. An observational study among patients with a high-grade squamous intraepithelial lesion (HSIL), who had LLETZ reported that HPV infection was not completely eradicated [35].

Two risk factors remained significantly associated with HPV infection after multivariate binomial regression analysis: marital and employment status. In a multivariate model, marital status was associated with reduced odds of hr-HPV infection. Single women may have a higher risk of HPV infection compared to married women due to risky sexual behavior such as not having a consistent sexual partner [42]. The data is consistent with previous studies that have reported a higher risk of HPV infection in single women [21, 22, 43]. Consistent with a previous study, unemployed women were significantly associated with hr-HPV infection [44]. This could be explained by the possibility that these women are low-income earners which could increase their level of poverty and high-risk sexual practices [44]. Contrasting findings were reported by Ginindza et al. [21] whereby there was no significant association between employment status and hr-HPV infection.

Furthermore, in a multivariate binomial regression model, only marital status (married vs. single) and employment status remained significantly associated with hr-HPV infection in women who attended routine Pap smear.

To our knowledge, no studies have investigated potential factors associated with hr-HPV E6/E7 mRNA expression in our setting.. Multivariate binomial regression analysis identified age as the most important risk factor for hr-HPV E6/E7 mRNA. Advanced age is an important factor linked to hr-HPV E6/E7 mRNA positivity. In a multivariate model, only age remained significantly associated with hr-HPV E6/E7 mRNA in women who attended routine Pap smear.

### Limitations

Hr-HPV E6/E7 mRNA could have been overestimated as only hr-HPV DNA-positive samples were tested. Hr-HPV DNA-positive samples were more likely to be hr-HPV E6/E7 mRNA positive than hr-HPV DNA-negative samples. Furthermore, the Abbott HR-HPV assay targets the L1 region which is often deleted when integration occurs during disease progression. Therefore, we may have missed samples that could be hr-HPV DNA-positive when excluding hr-HPV DNA-negative samples for hr-HPV E6/E7 mRNA testing. This could have caused underestimation. Technical aspects such as storage of the samples could have degraded the genetic material (i.e., mRNA is less stable than DNA). HIV status was not included in the study, given that South Africa has a high prevalence of HIV, it would have been interesting to determine if HIV infection had any significant effect on hr-HPV infection and hr-HPV E6/E7 mRNA expression in this population.

## Conclusions

The study found a high prevalence of hr-HPV infection and hr-HPV E6/E7 mRNA among women attending a tertiary hospital in Gauteng Province, South Africa. The high prevalence of hr-HPV E6/E7 mRNA indicates active HPV infection and thus a greater risk of developing cervical disease. The use of hr-HPV E6/E7 mRNA testing could identify women at greater risk and reduce unnecessary referrals, and subsequently the burden on healthcare facilities. Further research should be conducted to evaluate hr-HPV E6/E7 mRNA testing as a primary screening test, or co-testing together with liquid-based cytology, and/or follow up test for women already positive using cytology in the South African cervical cancer screening context.

## Supplementary Information

**Additional file 1**: **Table S1**. Factors associated with hr-HPV infection in women attending gynecology clinics for different reasons. **Table S2**. Factors associated with hr-HPV E6/E7 mRNA expression in women attending gynecology clinics for different reasons.

## Data Availability

The datasets used and analyzed during the current study are available from the corresponding author on reasonable request.

## Abbreviations

Cl: Confidence interval
DNA: Deoxyribonucleic acid
HIV: Human immunodeficiency virus
HPV: Human papilloma virus
HR: High-risk
IC: Internal control
LBC: Liquid-based cytology
LLETZ: Large loop excision of the transformation zone
LMIC: Low- and middle-income countries
LR: Low-risk
mRNA: Messenger ribonucleic acid
NDoH: National department of health
Pap: Papanicolaou
PR: Prevalence ratio
pRb: Retinoblastoma protein
SD: Standard deviation
STI: Sexually transmitted infection
TOP: Termination of pregnancy
