# Supplementary material for: High prevalence of and factors associated with human papillomavirus infection among women attending a tertiary hospital in Gauteng Province, South Africa
Source: BMC Cancer. 2022 Aug 5;22:854. doi: 10.1186/s12885-022-09964-9 (PMC9354293; doi:10.1186/s12885-022-09964-9)
Supplement: Supplementary file 1 — Additional file 1: Table S1. Factors associated with hr-HPV infection in women attending gynecology clinics for different reasons. Table S2. Factors associated with hr-HPV E6/E7 mRNA expression in women attending gynecology clinics for different reasons. [file 12885_2022_9964_MOESM1_ESM.docx]

Table S 1: Factors associated with hr-HPV infection in women attending gynecology clinics for different reasons

|  | **Routine Pap smear (n=232)** | | | | | | **LLETZ (n=87)** | | | | **Termination of Pregnancy (n=87)** | | | | **Review with PAP smear after LLETZ (n=57)4** | | | |
| --- | --- | --- | --- | --- | --- | --- | --- | --- | --- | --- | --- | --- | --- | --- | --- | --- | --- | --- |
|  | **Total** | **HR-HPV**  **prevalence** | **Bivariate analysis** |  | **Multivariate analysis** |  | **Total** | **HR-HPV prevalence** | **Bivariate analysis** |  | **Total** | **HR-HPV prevalence** | **Bivariate analysis** |  | **Total** | **HR-HPV prevalence** | **Bivariate analysis** |  |
|  | **n** | **n (%)** | **PR (95%CI)** | **p-value** | **PR (95%CI)** | **p-value** | **n** | **n (%)** | **PR (95%CI** | **p-value** | **n** | **n (%)** | **PR (95%CI** | **p-value** | **n** | **n (%)** | **PR (95%CI** | **p-value** |
| **Characteristics** |  |  |  |  |  |  |  |  |  |  |  |  |  |  |  |  |  |  |
| Age (years) | | | | | | | | | | | | | | | | | | |
| 18-29 | 50 | 27 (54.0) | 1.00 |  |  |  | 42 | 36 (85.7) | 1.00 |  | 66 | 38 (57.6) | 1.00 |  | 22 | 10 (45.5) | 1.00 |  |
| 30-39 | 77 | 24 (31.2) | 0.58 (0.38-0.88) | **0.010** |  |  |  |  |  |  | 18 | 8 (44.4) | 0.77 (0.44-1.35) | 0.36 |  |  |  |  |
| 40-49 | 56 | 16 (28.6) | 0.53 (0.33-0.86) | **0.010** |  |  | 30 | 25 (83.3) | 0.97 (0.79-1.19) | 0.78 |  |  |  |  | 26 | 14 (53.8) | 0.85 (0.48-1.49) | 0.56 |
| 50-59 | 35 | 7 (20.0) | 0.37 (0.18-0.75) | **0.006** |  |  | 15 | 8 (53.3) | 0.62 (0.38-1.01) | **0.057** |  |  |  |  | 9 | 3 (33.3) | 0.11 (0.67-2.23) | 0.51 |
| 60+ | 14 | 4 (28.6) | 0.53 (0.22-1.26) | 0.15 |  |  |  |  |  |  |  |  |  |  |  |  |  |  |
| Marital status | | | | | | | | | | | | | | | | | | |
| Single | 130 | 59 (45.4) | 1.00 |  |  |  | 65 | 51 (78.5) | 1.00 |  | 84 | 45 (53.6) | 1.00 |  | 34 | 20 (58.8) | 1.00 |  |
| Married | 74 | 13 (17.6) | 0.39 (0.23-0.66) | **0.0004** | 0.45 (0.27-0.75**)** | **0.0025** | 22 | 18 (81.8) | 1.04 (0.82-1.32) | 0.73 | 3 | 1 |  |  | 23 | 10 (43.5) | 0.74 (0.43-1.27) | 0.28 |
| Divorced/  Widowed/  Separated | 28 | 6 (21.4) | 0.47 (0.23-0.98) | **0.045** | 0.52 (0.26-1.08) | 0.078 |  |  |  |  |  | 0 |  |  |  |  |  |  |
| Employment status | | | | | | | | | | | | | | | | | | |
| Employed | 153 | 36 (23.5) | 1.00 |  |  |  | 25 | 22 (88.0) | 1.00 |  | 18 | 8 (44.4) | 1.00 |  | 18 | 7 (38.9) | 1.00 |  |
| Unemployed | 79 | 42 (53.2) | 2.26 (1.59-3.22) | **<.0001** | 1.99 (1.41-2.81) | **<0.0001** | 62 | 47 (75.8) | 0.86 (0.70-1.05) | 0.15 | 69 | 38 (55.1) | 1.24 (0.71-2.17) | 0.45 | 39 | 23 (59.0) | 1.52 (0.80-2.86) | 0.20 |
| Place of residence | | | | | | | | | | | | | | | | | | |
| Semi-urban | 197 | 71 (36.0) | 1.00 |  |  |  | 67 | 53 (79.1) | 1.00 |  | 81 | 43 (53.1) | 1.00 |  | 49 | 28 (57.1) | 1.00 |  |
| Semi-rural | 33 | 7 (21.2) | 0.59 | 0.13 |  |  | 20 | 16 (80.0) | 1.01 (0.79-1.30) | 0.93 | 4 | 3 |  |  | 8 | 2 |  |  |
| Rural |  |  |  |  |  |  |  |  |  |  |  |  |  |  |  |  |  |  |
| Number of children | | | | | | | | | | | | | | | | | | |
| None | 37 | 14 (37.8) | 1.00 |  |  |  | 57 | 47 (82.5) | 1.00 |  | 29 | 16 (55.2) | 1.00 |  | 36 | 19 (52.8) | 1.00 |  |
| 1 or 2 | 138 | 49 (35.5) | 0.94 (0.59-1.50) | 0.79 |  |  |  |  |  |  |  | 25 (54.3) | 0.99 (0.65-1.50) | 0.94 |  |  |  |  |
| 3 or 4 | 57 | 15 (26.3) | 0.70 (0.38-1.27) | 0.24 |  | | 30 | 22 (73.3) | 0.89 (0.69-1.17) |  | 12 | 5 (41.7) | 0.76 (0.3-1.59) | 0.46 | 21 | 11 (52.4) | 0.99 (0.60-1.66) | 0.98 |
| 5 and more |  |  |  |  |  |  |  |  |  |  |  |  |  |  |  |  |  |  |
| Present sexual activity status | | | | | | | | | | | | | | | | | | |
| Active | 183 | 61 (33.3) | 0.96 (0.62-1.48) | 0.86 |  |  | 62 | 49 (79.0) | 0.99 (0.78-1.25) | 0.92 | 73 | 37 (50.7) | 0.79 (0.50-1.24) |  | 47 | 24 (51.1) | 0.85 (0.48-1.52) | 0.58 |
| Not active | 49 | 17 (34.7) | 1.00 |  |  |  | 25 | 20 (80.0) | 1.00 |  | 14 | 9 (64.3) | 1.00 |  | 10 | 6 (60.0) | 1.00 |  |
| Number of current sexual partners | | | | | | | | | | | | | | | | | | |
| None | 45 | 16 (35.6) | 1.00 |  |  |  | 25 | 20 (80.0) | 1.00 |  | 11 | 7 (63.6) | 1.00 |  | 10 | 6 (60.0) | 1.00 |  |
| 1 | 187 | 62 (33.2) | 0.93 (0.60-1.45) | 0.76 |  | | 62 | 48 (79.0) | 0.99 (0.78-1.25) | 0.92 | 76 | 39 (51.3) | 0.81 (0.49-1.33) | 0.40 | 47 | 24 (51.1) | 0.85 (0.48-1.52) | 0.58 |
| 2 or more |  |  |  |  |  |  |  |  |  |  |  |  |  |  |  |  |  |  |
| Number of past sexual partners over 12 months | | | | | | | | | | | | | | | | | | |
| None | 44 | 15 (34.1) | 1.00 |  |  |  | 25 | 20 (80.0) | 1.00 |  | 12 | 6 (50.0) | 1.00 |  | 31 | 16 (51.6) | 1.00 |  |
| 1 | 107 | 32 (29.9) | 0.88 (0.53-1.45) | 0.61 |  |  | 22 | 17 (77.3) | 0.97 (0.72-1.30) | 0.82 | 42 | 20 (47.6) | 0.95 (0.50-1.82) | 0.88 |  |  |  |  |
| 2 | 35 | 15 (42.9) | 1.26 (0.72-2.20) | 0.42 |  |  | 10 | 8 (80.0) | 1.00 (0.69-1.44) | >0.99 | 15 | 10 (66.7) | 1.33 (0.68-2.60) | 0.40 | 12 | 8 (66.7) | 1.29 (0.76-2.18) | 0.34 |
| 3 | 26 | 8 (30.8) | 0.90 (0.44-1.83) | 0.78 |  |  | 15 | 13 (86.7) | 1.08 (0.82-1.43) | 0.57 | 18 | 10 (55.6) | 1.11 (0.55-2.24) | 0.77 |  |  |  |  |
| 4 or more | 20 | 8 (40.0) | 1.17 (0.60-2.31) | 0.64 |  |  | 15 | 11 (73.3) | 0.92 (0.64-1.32) | 0.4 |  |  |  |  | 14 | 6 (42.9) | 0.83 (0.41-1.66) | 0.60 |
| Use of contraceptives | | | | | | | | | | | | | | | | | | |
| Not using | 156 | 50 (32.1) | 1.00 |  |  |  | 60 | 47 (79.3)) |  |  | 79 | 43 (54.4) | 1.00 |  | 37 | 19 (51.4) | 1.00 |  |
| Using | 76 | 28 (36.8) | 1.15 (0.79-1.67) | 0.46 |  |  | 27 | 22 (81.5) |  |  | 8 | 3 |  |  | 20 | 11 (55.0) | 1.07 (0.65-1.78) | 0.79 |
| Period of contraceptive use | | | | | | | | | | | | | | | | | | |
| 6 months | 14 | 7 (50.0) | 1.56 (0.88-2.76) | 0.13 |  |  | 13 | 9 (69.2) | 0.98 (0.73-1.31) | 0.87 | 4 | 1 |  |  | 5 | 3 |  |  |
| 12 months | 13 | 6 (46.2) | 1.44 (0.77-2.70) | 0.26 |  |  |  |  |  |  |  |  |  |  |  |  |  |  |
| 24 months | 19 | 5 (26.3) | 0.82 (0.37-1.80) | 0.62 |  |  | 14 | 13 (92.9) | 1.15 (0.90-1.47) | 0.27 | 4 | 2 |  |  | 15 | 8 |  |  |
| more than 24 months | 30 | 10 (33.3) | 1.04 (0.60-1.81) | 0.89 |  |  |  |  |  |  |  |  |  |  |  |  |  |  |
| N/A | 156 | 50 (32.1) | 1.00 () |  |  |  | 60 | 47 (78.3) | 1.00 |  | 79 | 43 (54.4) | 1.00 |  | 37 | 19 (51.4) | 1.00 |  |
| Pregnant | | | | | | | | | | | | | | | | | | |
| Not pregnant | 223 | 75 (33.) | 1.00 |  |  |  | 84 | 66 (78.6) | 1.00 |  | 3 | 1 |  |  | 57 | 30 (52.6) | 1.00 |  |
| Pregnant | 4 | 1 |  |  |  |  |  |  |  |  | 83 | 45 (54.2) | 1.00 |  |  |  |  |  |
| Don’t know |  |  |  |  |  |  |  |  |  |  |  |  |  |  |  |  |  |  |

Table S 2: Factors associated with hr-HPV E6/E7 mRNA expression in women attending gynecology clinics for different reasons

|  | **Routine Pap smear (n=78)** | | | | | | **Termination of Pregnancy (n=46)** | | | |
| --- | --- | --- | --- | --- | --- | --- | --- | --- | --- | --- |
|  | **Total** | **HR-HPV**  **prevalence** | **Bivariate analysis** |  | **Multivariate analysis** |  | **Total** | **HR-HPV prevalence** | **Bivariate analysis** |  |
|  | **n** | **n (%)** | **PR (95%CI)** | **p-value** | **PR (95%CI)** | **p-value** | **n** | **n (%)** | **PR (95%CI** | **p-value** |
| **Characteristics** |  |  |  |  |  |  |  |  |  |  |
| Age (years) | | | | | | |  | | | |
| 18-29 | 27 | 17 (63.0) | 1.00 |  |  |  | 38 | 26 (68.4) | 1.00 |  |
| 30-39 | 24 | 16 (66.7) | 1.06 (0.71-1.59) | 0.78 | 1.00 | 0.78 | 8 | 8 |  |  |
| 40-49 | 16 | 15 (93.8) | 1.49 (1.09-2.04) | **0.014** | 1.06 (0.71-1.59) | **0.014** |  |  |  |  |
| 50-68 | 11 | 10 (90.9) | 1.44 (1.02-2.04) | **0.037** | 1.49 (1.09-2.04) | **0.037** |  |  |  |  |
| Marital status | | | | | | |  | | | |
| Single | 59 | 45 (76.3) | 1.00 |  |  |  | 45 | 33 (73.3) |  |  |
| Married | 19 | 13 (68.4) | 0.90 (0.64-1.26) | 0.53 |  |  | 1 | 1 |  |  |
| Divorced/Widowed/  Separated |  |  |  |  |  |  |  |  |  |  |
| Employment status | | | | | | |  | | | |
| Employed | 36 | 26 (72.2) | 1.00 |  |  |  | 8 | 6 | 1.00 |  |
| Unemployed | 42 | 32 (76.2) | 1.05 (0.81-1.37) | 0.69 |  |  | 38 | 28 (73.7) |  |  |
| Place of residence | | | | | | |  | | | |
| Semi-urban | 71 | 51 (71.8) | 1.00 |  |  |  | 43 | 32 (74.4) | 1.00 |  |
| Semi-rural/  Rural | 7 | 7 |  |  |  |  | 3 | 2 |  |  |
| Number of children | | | | | | |  | | | |
| None | 14 | 10 (71.4) | 1.00 |  |  |  | 16 | 11 (68.8) | 1.00 |  |
| 1 or 2 | 49 | 37 (75.5) | 1.06 (0.73-1.53) | 0.77 |  |  | 30 | 23 (76.7) | 1.12 (0.76-1.64) | 0.58 |
| 3 or 4 | 15 | 11 (73.3) | 1.03 (0.65-1.61) | 0.91 |  |  |  |  |  |  |
|  |  |  |  |  |  |  | 37 | 29 (78.4) |  |  |
| Present sexual activity status | | | | | | |  | | | |
| Active | 61 | 44 (72.1) | 0.88 (0.67-1.15) | 0.88 |  |  | 37 | 29 (73.9) |  |  |
| Not active | 17 | 14 (84.2) | 1.00 |  |  |  | 9 | 5 | 1.00 |  |
| Number of current sexual partners | | | | | | |  | | | |
| None | 16 | 14 (87.5) | 1.00 |  |  |  | 7 | 4 | 1.00 |  |
| 1 | 62 | 44 (71.0) | 0.81 (0.64-1.04) | **0.093** |  |  | 35 | 27 (77.1) |  |  |
| 2 or more |  |  |  |  |  |  | 4 | 3 |  |  |
| Number of past sexual partners over 12 months | | | | | | |  | | | |
| None | 15 | 13 (86.7) | 1.00 |  |  |  | 26 | 17 (65.4) | 1.00 |  |
| 1 | 32 | 22 (68.8) | 0.79 (0.58-1.08) | 0.14 |  |  |  |  |  |  |
| 2 | 15 | 11 (73.3) | 0.85 (0.59-1.22) | 0.37 |  |  | 10 | 9 (90.0) | 1.38 (0.97-1.95) | 0.40 |
| 3 | 16 | 12 (75.0) | 0.87 (0.611.22) | 0.41 |  |  | 10 | 8 (80.0) | 1.22 (0.81-1.86) | 0.77 |
| 4 or more |  |  |  |  |  |  |  |  |  |  |
| Use of contraceptives | | | | | | |  | | | |
| Not using | 50 | 36 (72.0) | 1.00 |  |  |  | 43 | 31 (72.1) | 1.00 |  |
| Using | 28 | 22 (78.6) | 1.09 (0.84-1.41) | 0.51 |  |  | 3 | 3 |  |  |
| Period of contraceptive use | | | | | | |  | | | |
| 6-24 months | 18 | 12 (66.7) | 0.95 (0.71-1.27) | 0.73 |  |  | 3 | 3 |  |  |
| More than 24 months | 10 | 10 (100.0) | Not est. |  |  |  | 0 | 0 |  |  |
| N/A | 50 | 36 (72.0) | 1.00 |  |  |  | 43 | 31 (72.1) | 1.00 |  |
|  |  |  |  |  |  |  |  |  |  |  |
| Not pregnant | 75 | 55 (73.3 | 1.00 |  |  |  | 1 | 1 |  |  |
| Pregnant | 1 | 1 |  |  |  |  | 45 | 33 (73.3) | 1.00 |  |
| Don’t know |  |  |  |  |  |  |  |  |  |  |
